# The Correlation between Severity of Neurological Impairment and Left Ventricular Function in Patients after Acute Ischemic Stroke

**DOI:** 10.3390/jcm8020190

**Published:** 2019-02-05

**Authors:** Pei-Hsun Sung, Kuan-Hung Chen, Hung-Sheng Lin, Chi-Hsiang Chu, John Y Chiang, Hon-Kan Yip

**Affiliations:** 1 Division of Cardiology, Department of Internal Medicine, Kaohsiung Chang Gung Memorial Hospital and Chang Gung University, Kaohsiung 83301, Taiwan; e12281@cgmh.org.tw; 2 Center for Shockwave Medicine and Tissue Engineering, Kaohsiung Chang Gung Memorial Hospital, Kaohsiung 83301, Taiwan; 3 Department of Anesthesiology, Kaohsiung Chang Gung Memorial Hospital and Chang Gung University College of Medicine, Kaohsiung 83301, Taiwan; amigotina@gmail.com; 4 Department of Neurology, Cognition and Aging Center, Kaohsiung Chang Gung Memorial Hospital and Chang Gung University College of Medicine, Kaohsiung 83301, Taiwan; a2522kimo@yahoo.com.tw; 5 Clinical Trial Center, Kaohsiung Chang Gung Memorial Hospital, Kaohsiung 83301, Taiwan; loveweib@gmail.com; 6 Quanzhou University of Information Engineering, Quanzhou 362000, China; chiang@mail.cse.nsysu.edu.tw; 7 Department of Computer Science & Engineering, National Sun Yat-sen University, Kaohsiung 80424, Taiwan; 8 Institute for Translational Research in Biomedicine, College of Medicine, Kaohsiung Chang Gung Memorial Hospital and Chang Gung University, Kaohsiung 83301, Taiwan; 9 Department of Medical Research, China Medical University Hospital, China Medical University, Taichung 40402, Taiwan; 10 Department of Nursing, Asia University, Taichung 41354, Taiwan

**Keywords:** acute ischemic stroke, left ventricular ejection fraction, inflammation, neutrophil-to-lymphocyte ratio, platelet-to-lymphocyte ratio, National Institute of Health Stroke Scale, modified Rankin scale

## Abstract

Despite left ventricular (LV) dysfunction increases the risk of incidental acute ischemic stroke (AIS), the association between LV function and severity of neurological deficits after AIS remains unclear. Between November 2015 and October 1017, a total of 99 AIS patients were prospectively enrolled and categorized into two groups based on National Institute of Health Stroke Scale (NIHSS). The AIS patients with NIHSS <6 were allocated into Group 1 (*n* = 50) and those with NIHSS ≥6 were into Group 2 (*n* = 49). Echocardiography was performed within 5 days after AIS to assess chamber size, left ventricular ejection fraction (LVEF) and valvular regurgitation. Besides, two inflammatory biomarkers, neutrophil-to-lymphocyte ratio (NLR) and platelet-to-lymphocyte ratio (PLR), were evaluated on admission. The results showed Group 2 had significantly higher value of NLR and PLR (all *p*-values < 0.01) but lower LVEF (*p* = 0.001) and frequency of mitral regurgitation (*p* = 0.021) than Group 1. The NIHSS and modified Rankin scale were significantly negatively correlated with LVEF, whereas both were significantly positively correlated with NLR and PLR (all *p*-values < 0.02). Multivariate analysis showed LVEF <65%, aging and inflammation were significantly associated with NIHSS ≥6 (all *p*-values < 0.01). In conclusion, the AIS patients with NIHSS ≥6 had lower LVEF but more clinically dominant mitral regurgitation and higher NLR and PLR compared to those with NIHSS <6.

## 1. Introduction

Acute ischemic stroke (AIS) is not only a severe disabling cerebrovascular event [1] but also has a great impact on patient’s life and socioeconomic burden [2]. Although AIS can be prevented by controlling relevant risk factors [3], its prevalence and incidence remain rising with aging and atherosclerotic process [4]. Besides, once the victims of AIS lose their golden time for thrombolytic or endovascular therapy, large infarcts with subsequent severe brain damage, especially in middle cerebral artery, would further lead to multiple organ dysfunction syndrome and unfavorable outcomes [5,6,7]. Hence, the brain infarct area/volume has been identified to be strongly correlated with the morbidity and mortality in patients after AIS [8].

The phenomenon of severe immune dysregulation, systemic inflammatory reaction and hematological suppression following acute severe brain injury has been found mediated by the signal pathway of damage-associated molecular patterns (DAMPs) [9]. The DAMPs and corresponding downstream signaling have been well studied to be involved in overwhelming inflammatory response, cytokine storm and profound immune perturbation [10,11,12]. Additionally, clinical observation study has previously revealed that 25% to 30% of the brain death patients had left ventricular (LV) dysfunction [13]. Furthermore, previous studies [14,15,16] have shown that the level of brain natriuretic peptide (BNP) or N-terminal pro-BNP (NT-proBNP) can be used for detection of cardioembolic stroke and is strongly related to severity of stroke. Data from our previous study [17] has also demonstrated that the NT-proBNP, a useful biomarker for predictive of acute decompensated heart failure, is not only significantly increased in patients after AIS but also an independent predictor for unfavorable neurological outcomes. These findings [13,14,15,16] raise the hypothesis that there may be a strongly negative correlation between the neurological functional status and the left ventricular ejection fraction (LVEF) in patients after AIS. However, relevant data to address this issue has been regrettably rarely reported [17].

On the other hand, it is well recognized that the crucial contributors to short-term and long-term moralities after AIS are the non-cerebrovascular complications such as severe infection, sepsis, myocardial infarction or other major organ (heart, lung or kidney) dysfunction, rather than cerebrovascular event per se. This issue may highlight the concept that “to improve neurological prognosis after AIS, the integrity of LV systolic function must be as early evaluated and preserved as possible.” With this concept in mind and the aforementioned issues [10,11,12,13,14,15,16,17], we, therefore, conducted a clinical study to investigate the correlation of LV ejection fraction (LVEF) with the stroke severity scores.

## 2. Materials and Methods

### 2.1. Study Design

This prospectively clinical study was conducted in a tertiary medical center of southern Taiwan from November 2015 to October 2017. The study protocol was approved by the institutional review boards (IRB number: 104-5222B) of Kaohsiung Chang Gung Memorial Hospital and written informed consents were obtained from all participants before enrollment.

### 2.2. Inclusion and Exclusion Criteria

Eligible patients aged between 45 and 80 years with AIS regardless of being treated by thrombolytic or endovascular therapy were consecutively and prospectively enrolled into this study. AIS was diagnosed by neurologists based upon detailed clinical assessment (Cincinnati Prehospital Stroke Scale) (Los Angeles Prehospital Stroke Screen), neurological examination (NE) and image modalities including brain computed tomography or magnet resonance imaging. The exclusion criteria included transient ischemic attack, young stroke, cerebellar infarcts, acute hemorrhagic stroke, traumatic brain injury, active infection without treatment, autoimmune diseases, malignancy with life expectancy less than one year, myocardial infarction less than one month, major surgery within three months, advanced liver cirrhosis and end-stage renal disease on peritoneal dialysis or hemodialysis. Besides, patients presenting with hemodynamic instability, post cardiopulmonary resuscitation or indication for immediate surgical intervention were also excluded from the study.

### 2.3. Definition of Stroke Severity

The quantification of stroke severity was performed by using National Institute of Health Stroke Scale (NIHSS) and Modified Rankin Scale (MRS) [18]. In addition, we evaluated the scales of neurologic deficit with NIHSS (0–42) within 12 hours of stroke and global disability severity with MRS (0–6). The patients with an NIHSS <6 are highly likely to have good clinical outcomes, whereas those with higher NIHSS expressed more severe stroke. Similarly, those with MRS of <3 are predicted to have an independent life after stroke. Therefore, we defined NIHSS ≥6 or MRS ≥3 as moderate to severe AIS.

### 2.4. Patients’ Enrollment and Allocation

Given previous clinical evidence showing [19] that the stroke patients with NIHSS ≥6 expressed moderate to severe neurological deficit with a need of admission to neurology intensive care unit (NICU) and had poorer clinical outcome as compared to those with NIHSS <6, we divided study subjects into two groups according to severity of AIS, that is, Groups 1 (NIHSS <6) and 2 (NIHSS ≥6). A sample size of 54 study subjects in each group was estimated based on two-tailed anticipated LVEF of 60% ± 8% versus 65% ± 8% between two groups while considering 20% rate of protocol violations and incomplete follow-up, with the setting of effective size = 0.625, alpha = 0.05 and power = 0.8. Between November 2015 and October 2017, a total of 110 consecutive subjects who met the inclusion and exclusion criteria were prospectively enrolled into the study. We further excluded 11 cases with hemorrhagic transformation (*n* = 4), life-threatening stress ulcer bleeding (*n* = 3), concomitant heart attack (*n* = 1), complications of aortic dissection (*n* = 1) and hospital transfer (*n* = 2) after enrollment. Finally, we assigned 99 AIS patients into Group 1 (*n* = 50) and Group 2 (*n* = 49) according to NIHSS ≥6 or not. All patients were completely surveyed during hospitalization and objectively assessed for in-hospital laboratory and clinical outcomes.

### 2.5. Laboratory Test for Circulatory Complete Blood Count/Differential Count

The red blood cell count, white blood cell count and platelet count as well as percentages of neutrophils and lymphocytes were routinely measured upon presentation by laboratory standard method. Additionally, for assessment of the correlation between neurological dysfunction and inflammatory reaction, the neutrophil-to-lymphocyte ratio (NLR) and platelet-to-lymphocyte ratio (PLR), two inflammatory parameters, were calculated in the present study.

### 2.6. Primary and Secondary Endpoints

We hypothesized that those patients with higher scores of NIHSS or MRS had a lower LVEF, so the primary endpoint was the correlation between LVEF and NIHSS or MRS, respectively. The secondary endpoints were to study the association between inflammatory indices and stroke severity and further identify the independent predictors for moderate to severe stroke. Besides, in-hospital clinical outcomes, including death and lung edema, were also evaluated between two groups.

### 2.7. Study Protocol for Evaluation and Clinical Follow-Up

Once AIS was diagnosed, the patient with mild AIS in Group 1 (NIHSS <6) were admitted to neurological ward for early treatment as guideline recommended [20]. On the other hand, those with moderate to severe AIS in Group 2 (NIHSS ≥6) were admitted to NICU for close monitoring of vital signs, hemodynamics, respiratory condition and neurological status. The treatment for stroke and underlying diseases were based upon the practice guidelines. Whether further management with ventilatory support or monitoring of intracranial pressure or not depended on the clinical situation. The patients’ information including baseline profile and comorbidities was available from patients’ or family’s statements, previous medical records or relevant clinical evidence on admission. Besides, all laboratory data comprising hemogram and biochemistry were acquired at emergency department upon presentation and just admitted to ward or NICU. Echocardiographic study was performed by a cardiologist blinded to the severity of NIHSS/MRS within five days after admission. Results from aforementioned laboratory and echocardiographic analyses were entered into computerized case profiles by a study nurse or research assistant blinded to assignment of groups. All clinical adverse events were acquired according to medical or nursing records.

### 2.8. Medications for AIS

Aspirin was prescribed for all AIS patients unless contraindicated. If intolerant or allergic to aspirin, clopidogrel was prescribed instead. As for those with cardioembolic stroke resulted from atrial fibrillation (AF), warfarin or direct oral anticoagulant was prescribed at appropriate time after stabilized neurological presentation according to NIHSS scores [21]. Other comorbidities or underlying diseases were treated with guideline-direct medications, including statins, oral antidiabetic agents, angiotensin-converting enzyme inhibitors/angiotensin II type I receptor blockers, diuretics, calcium channel blockers and beta blockades.

### 2.9. Echocardiographic Measurement for LV Systolic Function and Grade of Valvular Regurgitation

All subjects in either wards or NICU received echocardiographic study within 5 days after stroke. To evaluate cardiac chamber size, LVEF and grade of mitral regurgitation (MR) and tricuspid regurgitation (TR), conventional echocardiography was performed with standard 2-dimenional (2D) views, M-mode, tissue and color Doppler assessment. Digital images were collected and data were analyzed according to the standardized protocol [22]. Cardiologists who performed echo study were blinded to study allocation. Cardioprotective drugs were also adjusted in time according to abnormal findings.

### 2.10. Statistical Analysis

Independent t and Mann-Whitney u tests were used to compare the difference between groups for continuous variables as appropriate. For discrete or categorical variables, chi-square and Fisher exact tests were applied to detect the proportions between groups. Additionally, Pearson’s or Spearman’s correlation analysis was adopted to assess the relationship of NIHSS or MRS to LVEF, NLR and PLR. Area under the curve (AUC) of receiver operating characteristic (ROC) curve and Youden’s index were further used for calculation of cutoff value from moderate to severe AIS which was defined as NIHSS ≥6 or MRS ≥3. Finally, we performed logistic regression model with univariate and multivariate analyses to identify potential independent predictors for NIHSS ≥6 and MRS ≥3, respectively. Statistical analysis was performed using SPSS statistical software for Windows version 22 (SPSS for Windows, version 22; SPSS Inc., Chicago, IL, USA). A value of *p* < 0.05 was considered statistically significant.

## 3. Results

### 3.1. Patients’ Characteristics (Table 1)

The baseline variables demonstrated that there were no significant differences between Group 1 and Group 2 in terms of systolic and diastolic blood pressure and incidences of gender, hypertension, diabetes mellitus, old stroke, old myocardial infarction and utilization of ACEI or ARB. However, the age was older in Group 2 than in Group 1 patients. Additionally, the frequencies of smoking, dyslipidemia and statin prescription were significantly lower in Group 2 than Group 1.

The laboratory findings showed that the leukocyte and platelet counts, hemoglobin and percentage of neutrophils did not differ between these two groups of the patients. NLR and PLR were significantly higher, whereas the percentage of lymphocytes was significantly lower in Group 2 than in Group 1. The serum levels of creatinine, total cholesterol, LDL and HDL were similar between Group 1 and group 2, whereas the serum level of triglyceride was significantly higher in Group 1 than in Group 2. Furthermore, the average NIHSS and MRS were significantly increased in Group 2 than in Group 1.

**Table 1:** Baseline characteristics of the stroke patients between Groups 1 (NIHSS <6) and 2 (NIHSS ≥6).

Variables	Total (*n* = 99)	Group 1 (*n* = 50)	Group 2 (*n* = 49)	*p*-Value
Clinical				
Age/year	64.16 ± 11.85	60.74 ± 11.83	68.06 ± 10.51	0.002
Male sex/No. (%)	59 (59.6%)	30 (60.0%)	29 (59.2%)	0.934
Smoker/No. (%)	32 (32.3%)	21 (42.0%)	11 (22.4%)	0.038
Systolic BP/mmHg	161.92 ± 29.16	165.10 ± 30.76	158.67 ± 27.37	0.275
Diastolic BP/mmHg	88.99 ± 17.26	91.70 ± 16.19	86.22 ± 18.03	0.115
Hypertension/No. (%)	79 (79.8%)	38 (76.0%)	41 (83.7%)	0.324
Diabetes/No. (%)	34 (34.3%)	19 (38.0%)	15 (30.6%)	0.439
Dyslipidemia/No. (%)	45 (45.5%)	29 (58.0%)	16 (32.7%)	0.011
Old MI/No. (%)	3 (3.0%)	1 (2.0%)	2 (4.1%)	0.492
Old stroke/No. (%)	19 (19.2%)	8 (16.0%)	11 (22.4%)	0.415
Atrial fibrillation/No. (%)	11 (11.1%)	1 (2.0%)	10 (20.4%)	0.004
ACEI or ARB/No. (%)	47 (47.5%)	24 (48.0%)	23 (46.9%)	0.916
Statin/No. (%)	53 (53.5%)	33 (66.0%)	20 (40.8%)	0.012
Laboratory data				
Leukocyte count/1000/μL	8.38 ± 2.71	8.26 ± 2.78	8.51 ± 2.67	0.654
Neutrophil/1000/μL	5.39 (3.70–7.16)	4.97 (3.45–7.04)	5.80 (4.10–7.25)	0.136
Lymphocyte/1000/μL	1.93 (1.33–2.57)	2.12 (1.58–2.91)	1.45 (1.12–2.27)	0.001
Hemoglobin/g/dL	14.20 ± 2.04	14.40 ± 1.86	14.00 ± 2.21	0.329
Platelet count/1000/μL	212.14 ± 68.62	218.40 ± 79.74	205.76 ± 55.15	0.362
NLR	2.85 (1.67–4.66)	2.20 (1.45–3.72)	3.73 (2.27–5.72)	0.001
PLR	113.0 (81.3–155.4)	99.4 (77.4–125.6)	133.0 (93.8–178.7)	0.005
Serum creatinine/mg/dL	0.93 (0.75–1.22)	0.93 (0.72–1.18)	0.93 (0.78–1.47)	0.448
Total Cholesterol/mg/dL	180.0 (153.3–210.5)	179.5 (160.8–210.5)	181.0 (147.3–210.3)	0.779
HDL/mg/dL	43.71 ± 13.00	44.04 ± 15.32	43.38 ± 10.20	0.802
LDL/mg/dL	104.62 ± 46.64	101.42 ± 45.42	107.96 ± 48.13	0.491
Triglyceride/mg/dL	113.0 (78.5–166.5)	135.5 (91.8–188.3)	98.5 (65.8–139.8)	0.004
Stroke severity				
Average NIHSS	8.59 ± 8.45	2.74 ± 1.40	14.55 ± 8.47	<0.001
Modified Rankin Scale	2.87 ± 1.50	1.92 ± 1.05	3.84 ± 1.25	<0.001

Data are expressed as means ± standard deviation, median (1st quantile to 3rd quantile) or No. (%). Abbreviation: NIHSS, National Institute of Health Stroke Scale; BP, blood pressure; MI, myocardial infarction; ACEI, angiotensin-converting-enzyme inhibitor; ARB, angiotensin II receptor blocker; NLR, neutrophil-to-lymphocyte ratio; PLR, platelet-to-lymphocyte ratio; HDL, high-density lipoprotein; LDL, low-density lipoprotein.

### 3.2. Echocardiographic Results and Clinical Outcomes (Table 2)

The results of echocardiographic study showed a significantly lower LVEF in Group 2 than Group 1. Additionally, patients with NIHSS ≥6 had significantly higher prevalence of obvious MR, whereas the left ventricular end-systolic diameter exhibited an opposite pattern of LVEF between the two groups, suggesting that the more severe AIS was, the more obvious LV systolic and mitral valve dysfunctions were. On the other hand, the incidences of in-hospital mortality and lung edema, frequency of tricuspid regurgitation, as well as LV end diastolic diameter did not differ between Group 1 and Group 2 patients.

**Table 2:** Echocardiographic and clinical outcomes between groups 1 (NIHSS <6) and 2 (NIHSS ≥6).

Variables	Total (*n* = 99)	Group 1 (*n* = 50)	Group 2 (*n* = 49)	*p*-Value
Echocardiographic data				
IVS thickness/mm	11.14 ± 4.55	11.47 ± 5.86	10.08 ± 2.75	0.815
LVPW thickness/mm	8.82 ± 1.76	8.82 ± 1.79	8.83 ± 1.75	0.758
LVEDD/mm	48.89 ± 8.08	47.30 ± 7.03	50.52 ± 8.82	0.054
LVESD/mm	31.71 ± 8.06	29.94 ± 7.27	33.71 ± 8.42	0.009
LVEF/%	64.96 ± 10.93	68.57 ± 7.84	61.27 ± 12.41	0.001
Mild to severe MR/No. (%)	26 (28.3%)	8 (17.4%)	18 (39.1%)	0.021
Mild to severe TR/No. (%)	23 (25.0%)	9 (19.6%)	14 (30.4%)	0.229
Mitral stenosis/No. (%)	0 (0%)	0 (0%)	0 (0%)	-
Aortic stenosis/No. (%)	0 (0%)	0 (0%)	0 (0%)	-
Clinical outcomes				
In-hospital death/No. (%)	2 (2.0%)	0 (0.0%)	2 (4.1%)	0.242
Pulmonary edema/No. (%)	1 (1.0%)	0 (0.0%)	1 (2.0%)	0.495

Data are expressed as means ± standard deviation, median (1st quantile to 3rd quantile) or No. (%). Abbreviation: NIHSS, National Institute of Health Stroke Scale; IVS, interventricular septum; LVPW, left ventricular posterior wall; LVEDD, left ventricular end-diastolic diameter; LVESD, left ventricular end-systolic diameter; LVEF, left ventricular ejection fraction; MR, mitral regurgitation; TR, tricuspid regurgitation.

### 3.3. The Relationship of Stroke Severity (NIHSS or MRS) to Inflammatory Markers (NLR or PLR) and LV Systolic Function, followed by Identifying Cutoff Values of Moderate to Severe Stroke (Table 3 and Figure 1)

Correlation analysis showed a very significantly high correlation (*R* = 0.775) between NIHSS and MRS, index for severity of AIS and subsequent neurological impairment, respectively. Besides, both NIHSS and MRS were also significantly positively correlated with NLR or PLR, whereas these two parameters showed a significantly negative correlation with LVEF (all *p*-values < 0.02), indicating that patients with more severe stroke had poorer LV systolic function and higher systemic inflammatory reaction.

**Table 3:** Correlation of NLR, PLR and LV systolic function to stroke severity (MRS and NIHSS).

Variables	Correlation Coefficient (*R*)	*p*-Value
NIHSS vs. MRS	0.775	<0.001
NIHSS vs. NLR	0.353	<0.001
NIHSS vs. PLR	0.269	0.007
NIHSS vs. LVEF	−0.369	<0.001
MRS vs. NLR	0.284	0.004
MRS vs. PLR	0.237	0.018
MRS vs. LVEF	−0.250	0.016

Abbreviation: NLR, neutrophil-to-lymphocyte ratio; PLR, platelet-to-lymphocyte ratio; LVEF, left ventricular ejection fraction; NIHSS, National Institute of Health Stroke Scale; MRS, Modified Rankins Scale; R, Pearson’s or Spearman’s correlation coefficient.

**Figure 1:**
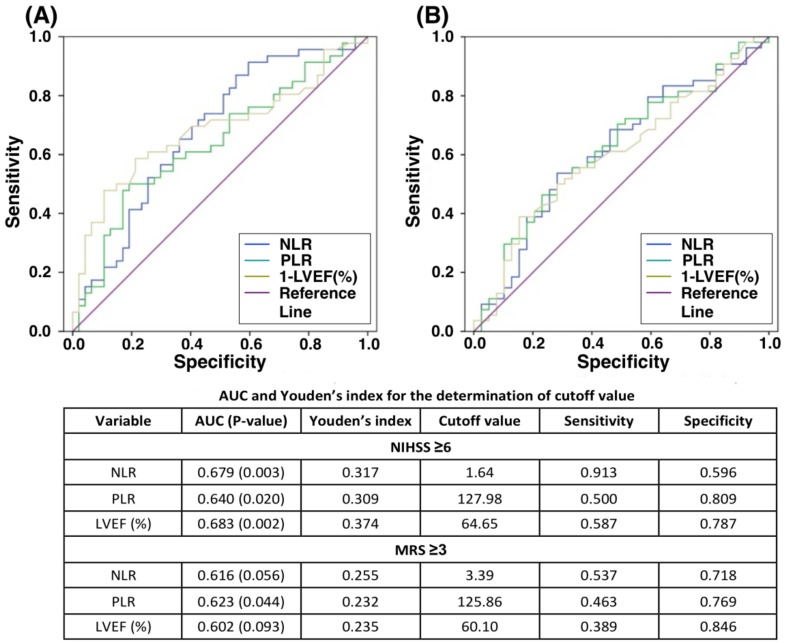
ROC curve for the correlation of NLR, PLR and LVEF to NIHSS (A) or MRS (B). (**A**) ROC curve reveals NLR, PLR and LVEF had mild correlation to NIHSS ≥6, with significantly acceptable discrimination power of AUC (0.640–0.683) for all three parameters. (**B**) Correlation of NLR, PLR and LVEF with MRS ≥3 were mild and only PLR exhibited significantly mild discrimination power of AUC (0.623, *p* = 0.004). Abbreviation: AUC, area under the receiver operating characteristic (ROC) curve; NIHSS, National Institute of Health Stroke Scale; MRS, Modified Rankin Scale; NLR, neutrophil-to-lymphocyte ratio; PLR, platelet-to-lymphocyte ratio; LVEF, left ventricular ejection fraction.

ROC curve on Figure 1 shows that NLR, PLR and LVEF had an acceptable probability of detection for high scores of NIHSS or MRS. The cutoff values of NLR, PLR and LVEF for NIHSS ≥6 were 1.6, 128 and 65%, respectively (all *p*-values for AUC ≤0.02). Similarly, the threshold values of NLR, PLR and LVEF for MRS ≥3 were 3.4, 126 and 60%, respectively, although statistical power was not significant. Overall, the results suggested those patients with LVEF <60% might have more severe stroke with a need of ICU admission and predictable poorer neurological outcomes, especially combining NIHSS score ≥6 as a determinant.

### 3.4. Further Identification of Independent Risk Factors for Stroke Severity NIHSS ≥6 (Table 4)

Logistic regression analysis was performed to identify the potential predictors for those with moderate or severe stroke severity. Results of multivariate analysis revealed age >65 years and NLR ≥1.65 were two most powerful predictors of moderate to severe AIS. On the contrary, dyslipidemia and LVEF ≥65% were recognized to be significantly and conversely associated with severe neurological dysfunction. The similar findings were also observed by using MRS ≥3 as an index of moderate neurological impairment, except for LVEF, NLR, PLR or even combined PLR + PLR (ref. Table S1). These findings implied that not only serum inflammatory markers could be applied for prediction of moderate to severe stroke with NIHSS ≥6 but also deterioration of LV systolic function might be a useful parameter for predictive of more severe neurological function in patients after AIS.

**Table 4:** Predictors for stroke severity with NIHSS ≥6.

Stroke Severity	Univariate			Multivariate		
Variables	OR	95% CI	*p*-Value	OR	95% CI	*p*-Value
Age per year	1.061	1.020–1.102	0.003			
Age >65 years	4.004	1.735–9.237	0.001	4.400	1.560–12.415	0.005
Male sex	0.967	0.433–2.158	0.934			
Smoker	0.400	0.167–0.959	0.040			
Systolic BP	0.992	0.919–1.006	0.273			
Diastolic BP	0.981	0.958–1.005	0.118			
Hypertension	1.618	0.597–4.389	0.344			
Diabetes	0.720	0.313–1.657	0.440			
Dyslipidemia	0.351	0.155–0.797	0.012	0.280	0.097–0.813	0.019
Old MI	2.085	0.183–23.769	0.554			
Old stroke	1.520	0.553–4.176	0.417			
Atrial fibrillation	12.564	1.541–102.417	0.018			
ACEI or ARB	0.958	0.435–2.110	0.916			
Statin	0.355	0.157–0.804	0.013			
Leukocyte count	1.034	0.893–1.198	0.651			
Hemoglobin	0.906	0.743–1.104	0.328			
Platelet count	0.997	0.991–1.003	0.360			
NLR	1.202	1.024–1.411	0.024			
NLR ≥1.64	7.500	2.331–24.113	0.001	6.953	1.710–28.264	0.007
PLR	1.003	0.999–1.008	0.118			
PLR ≥128	4.522	1.854–11.029	0.001			
NLR ≥1.64 and PLR >128	4.552	1.854–11.029	0.001			
Serum creatinine	1.518	0.872–2.642	0.140			
Total Cholesterol	0.998	0.990–1.006	0.634			
HDL	0.996	0.966–1.027	0.799			
LDL	1.003	0.994–1.012	0.487			
Triglyceride	0.989	0.982–0.996	0.003			
IVS thickness	0.966	0.878–1.064	0.485			
LVEF per %	0.930	0.886–0.975	0.003			
LVEF ≥65%	0.190	0.076–0.474	<0.001	0.216	0.074–0.637	0.005
Mild to severe MR	3.054	1.163–8.018	0.023			

Abbreviation: NIHSS, National Institute of Health Stroke Scale; OR, odds ratio; CI, confidence interval; BP, blood pressure; MI, myocardial infarction; ACEI, angiotensin-converting-enzyme inhibitor; ARB, angiotensin II receptor blocker; NLR, neutrophil-to-lymphocyte ratio; PLR, platelet-to-lymphocyte ratio; HDL, high-density lipoprotein; LDL, low-density lipoprotein; IVS, interventricular septum; LVEF, left ventricular ejection fraction; MR, mitral regurgitation.

### 3.5. Subgroup Analysis According to AIS Patients with or without AF (Ref. to Table S2)

The results in Table S2 showed that AF patients expressed significantly higher stroke severity and more severe neurologic deficits than in non-AF patients. Additionally, the LVEF was also significantly lower in the AF than non-AF patients, whereas the NLR and PLR showed no difference between these two groups of patients. The findings from this subgroup analysis were consistent with our finding in the present study that severe stroke was associated with lower LVEF.

## 4. Discussion

This prospective study designed to investigate the correlation between LV systolic function and stroke severity yielded several striking findings. First, as compared to those with mild AIS, that is, NIHSS <6, patients with moderate to severe AIS, that is, NIHSS ≥6, had significantly lower LVEF and were older. Second, NLR and PLR, two indices of inflammation, were also significantly higher in those with NIHSS ≥6 than the NIHSS <6 counterparts. Third, NIHSS or MRS was significantly negatively correlated with LVEF but positively with NLR and PLR. Finally, our study found that lower LVEF and NLR >2 were strongly associated with moderate to severe stroke.

Numerous studies have shown that chronic LV systolic dysfunction or acute heart failure increases the risk of incidental AIS through sharing common traditional or nontraditional atherosclerotic risk factors [23,24,25]. Additionally, impaired LV systolic or diastolic functions have been identified as a powerful predictor for post-apoplectic poor neurologic outcomes or increased future vascular events [26,27]. Of the most importance was that the lower LVEF along with older age was significantly correlated to the severity of neurological dysfunction. Our finding, in addition to extending the findings of previous studies [23,24,25,26,27], could explain why non-cerebrovascular complications are the major contributors to long-term morbidity and mortality in patients after AIS and highlight that lower LVEF is a useful parameter for predictive of poor prognostic outcome in setting of AIS.

Interestingly, previously clinical study has revealed more than 25% of AIS patients have lower LVEF, even at the early stage of brain death [13]. Undoubtedly, an extremely severe neurological dysfunction, an indicator of severe neuron/brain damage, could be viewed as an equivalence of brain death. Accordingly, this scientific rationale and clinical observation from the previous research [13], at least in part, support the finding of our study.

An essential finding in the present study was that neurological severity was strongly correlated to the NLR and PLR, that is, two biomarkers representative of systemic inflammation. Intriguingly, our previous study [28] has displayed that NLR was independently associated with in-hospital mortality and higher neutrophil count was independently predictive of severe stroke. Thus, this important finding was supported by our previous study results [28]. Also, our recent experimental study [29] has shown that brain death caused remote organ damage and deterioration of heart function, mainly through DAMP signaling pathway. Taken together, the present finding supported by our two recent publications [12,29] could partially explain why the LVEF was more impaired in the AIS patients with high neurological deficit.

This was the first clinical observational study to demonstrate a positive relationship between LVEF and NIHSS or MRS. The findings indicated that LVEF not only could reflect stroke severity beyond traditional neurological scoring system such as NIHSS but also had an association with post-stroke neurological disability like MRS. Furthermore, new inflammatory biomarkers checked early upon presentation of AIS, that is, NLR and PLR, were also strongly associated with stroke severity. In clinical practice, multidisciplinary assessment with combination of LV systolic function, inflammatory parameters and traditional neurological scales for AIS helped clinicians easily and quickly identify patients with relatively severe stroke. Particularly, before expert consultation, general physicians were able to objectively recognize "potentially high-risk” AIS patients with a need of NICU setting early by performing focused NE, PLR/NLR checking and LVEF assessment.

It is well known that the pathophysiology of heart failure is characterized by hemodynamic and heart rate-variability abnormalities that result in neurohormonal activation and autonomic imbalance with increase in sympathetic tone and withdrawal of parasympathetic (i.e., vagal) activity [30]. Plentiful data have shown that disturbances of the autonomic nervous system are common in patients with various cerebrovascular diseases, especially in those of AIS patients [31]. Additionally, other studies have also established that autonomic and cardiac dysfunction frequently occur after vascular brain injury without any evidence of primary heart disease [32]. During acute stroke, autonomic dysfunction which is characterized by elevated arterial blood pressure, arrhythmia and ischemic cardiac damage has been clearly identified [30,32], which may hinder the prognostic outcome. Furthermore, an increased in NIHSS values predict impairment of cardiovascular autonomic control in setting of AIS [33]. In this way, all the autonomic changes may put patients with more severe stroke at increasing risk of cardiovascular complications and poor outcome [33]. These aforementioned evidences [30,31,32,33] may, at least in part, support the findings of the present study that LV dysfunction was strongly correlated with the severity of NIHSS.

This study has limitations. Frist, owing to lack of long-term follow-up for our patients, we did not know whether the LVEF is a useful predictor for the long-term prognostic outcome in patients after AIS. Second, the exact underling mechanism for why the more severe neurological defect after AIS was associated with lower LVEF remains unclear. Third, the sample size of the present study was relatively small. Thus, we did not completely exclude that the statistical significance would be distorted in some variable comparisons due to the small sample size. Fourth, without route examination of coronary angiographic study or thallium scan for those of AIS patients, we did not know how many percentages of the patients had obstructive coronary artery disease that would be associated with the lower LVEF. Finally, we did not routinely measure the circulating levels of BNP and high-sensitivity troponin. Hence, the frequency of elevation of these two biomarkers in AIS patients remains uncertain.

## 5. Conclusions

The present study demonstrated that AIS patients with higher neurological dysfunction not only were older but also had lower LVEF, more MR and higher NLR or PLR compared to those with lower NIHSS scores.

## Supplementary Materials

The following are available online at http://www.mdpi.com/2077-0383/8/2/190/s1, Table S1: Predictors for neurologic impairment with MRS ≥3, Table S2: Subgroup analysis according to AIS patients with or without AF.

## Abbreviations

AIS	acute ischemic stroke
DAMP	damage-associated molecular patterns
NLR	neutrophil-to-lymphocyte ratio
PLR	platelet-to-lymphocyte ratio
BNP	brain natriuretic peptide
NT-proBNP	N-terminal pro-BNP
LV	left ventricular
LVEF	left ventricular ejection fraction
NE	neurological examination
NIHSS	National Institute of Health Stroke Scale
MRS	modified Rankin scale
NICU	neurology intensive care unit
AF	atrial fibrillation
MR	mitral regurgitation
TR	tricuspid regurgitation
AUC	area under the curve
ROC curve	receiver operating characteristic curve

## References

[1] Donnan G.A., Fisher M., Macleod M., Davis S.M. (2008). Stroke. Lancet.

[2] Adams H.P., del Zoppo G., Alberts M.J., Bhatt D.L., Brass L., Furlan A., Grubb R.L., Higashida R.T., Jauch E.C., Kidwell C. (2007). Guidelines for the early management of adults with ischemic stroke: A guideline from the American Heart Association/American Stroke Association Stroke Council, Clinical Cardiology Council, Cardiovascular Radiology and Intervention Council and the Atherosclerotic Peripheral Vascular Disease and Quality of Care Outcomes in Research Interdisciplinary Working Groups: The American Academy of Neurology affirms the value of this guideline as an educational tool for neurologists. Stroke.

[3] D’Agostino R.B., Wolf P.A., Belanger A.J., Kannel W.B. (1994). Stroke risk profile: Adjustment for antihypertensive medication. The Framingham Study. Stroke.

[4] Ovbiagele B., Nguyen-Huynh M.N. (2011). Stroke epidemiology: Advancing our understanding of disease mechanism and therapy. Neurotherapeutics.

[5] Qureshi A.I., Suarez J.I., Yahia A.M., Mohammad Y., Uzun G., Suri M.F., Zaidat O.O., Ayata C., Ali Z., Wityk R.J. (2003). Timing of neurologic deterioration in massive middle cerebral artery infarction: A multicenter review. Crit. Care Med..

[6] Bill O., Zufferey P., Faouzi M., Michel P. (2013). Severe stroke: Patient profile and predictors of favorable outcome. J. Thromb. Haemost..

[7] Hacke W., Schwab S., Horn M., Spranger M., De Georgia M., von Kummer R. (1996). ‘Malignant’ middle cerebral artery territory infarction: Clinical course and prognostic signs. Arch. Neurol..

[8] Heinsius T., Bogousslavsky J., Van Melle G. (1998). Large infarcts in the middle cerebral artery territory. Etiology and outcome patterns. Neurology.

[9] Liesz A., Dalpke A., Mracsko E., Antoine D.J., Roth S., Zhou W., Yang H., Na S.Y., Akhisaroglu M., Fleming T. (2015). DAMP signaling is a key pathway inducing immune modulation after brain injury. J. Neurosci..

[10] Seong S.Y., Matzinger P. (2004). Hydrophobicity: An ancient damage-associated molecular pattern that initiates innate immune responses. Nat. Rev. Immunol..

[11] Janeway C. (1989). Immunogenicity signals 1,2,3 … and 0. Immunol. Today.

[12] Sung P.H., Lee F.Y., Lin L.C., Chen K.H., Lin H.S., Shao P.L., Li Y.C., Chen Y.L., Lin K.C., Yuen C.M. (2018). Melatonin attenuated brain death tissue extract-induced cardiac damage by suppressing DAMP signaling. Oncotarget.

[13] Bulcao C.F., D’Souza K.M., Malhotra R., Staron M., Duffy J.Y., Pandalai P.K., Jeevanandam V., Akhter S.A. (2010). Activation of JAK-STAT and nitric oxide signaling as a mechanism for donor heart dysfunction. J. Heart Lung Transplant..

[14] Chen X., Zhan X., Chen M., Lei H., Wang Y., Wei D., Jiang X. (2012). The prognostic value of combined NT-pro-BNP levels and NIHSS scores in patients with acute ischemic stroke. Intern. Med..

[15] Bai J., Sun H., Xie L., Zhu Y., Feng Y. (2018). Detection of cardioembolic stroke with B-type natriuretic peptide or N-terminal pro-BNP: A comparative diagnostic meta-analysis. Int. J. Neurosci..

[16] Wei W., Chen Y., Lei D., Zhang Y., Weng X., Zhou Y., Zhang L. (2018). Plasma brain natriuretic peptide is a biomarker for screening ischemic cerebral small vessel disease in patients with hypertension. Medicine.

[17] Yip H.K., Sun C.K., Chang L.T., Chen M.C., Liou C.W. (2006). Time course and prognostic value of plasma levels of N-terminal pro-brain natriuretic peptide in patients after ischemic stroke. Circ. J..

[18] Lyden P.D., Lu M., Levine S.R., Brott T.G., Broderick J. (2001). A modified National Institutes of Health Stroke Scale for use in stroke clinical trials: Preliminary reliability and validity. Stroke.

[19] Rost N.S., Bottle A., Lee J.M., Randall M., Middleton S., Shaw L., Thijs V., Rinkel G.J., Hemmen T.M. (2016). Stroke Severity Is a Crucial Predictor of Outcome: An International Prospective Validation Study. J. Am. Heart Assoc..

[20] Jauch E.C., Saver J.L., Adams H.P., Bruno A., Connors J.J., Demaerschalk B.M., Khatri P., McMullan P.W., Qureshi A.I., Rosenfield K. (2013). Guidelines for the early management of patients with acute ischemic stroke: A guideline for healthcare professionals from the American Heart Association/American Stroke Association. Stroke.

[21] Hankey G.J., Norrving B., Hacke W., Steiner T. (2014). Management of acute stroke in patients taking novel oral anticoagulants. Int. J. Stroke.

[22] Galderisi M., Henein M.Y., D’Hooge J., Sicari R., Badano L.P., Zamorano J.L., Roelandt J.R. (2011). Recommendations of the European Association of Echocardiography: How to use echo-Doppler in clinical trials: Different modalities for different purposes. Eur. J. Echocardiogr..

[23] Hays A.G., Sacco R.L., Rundek T., Sciacca R.R., Jin Z., Liu R., Homma S., Di Tullio M.R. (2006). Left ventricular systolic dysfunction and the risk of ischemic stroke in a multiethnic population. Stroke.

[24] Cuadrado-Godia E., Ois A., Roquer J. (2010). Heart failure in acute ischemic stroke. Curr. Cardiol. Rev..

[25] Haeusler K.G., Laufs U., Endres M. (2011). Chronic heart failure and ischemic stroke. Stroke.

[26] Ois A., Gomis M., Cuadrado-Godia E., Jimenez-Conde J., Rodriguez-Campello A., Bruguera J., Molina L., Comin J., Roquer J. (2008). Heart failure in acute ischemic stroke. J. Neurol..

[27] Park H.K., Kim B.J., Yoon C.H., Yang M.H., Han M.K., Bae H.J. (2016). Left Ventricular Diastolic Dysfunction in Ischemic Stroke: Functional and Vascular Outcomes. J. Stroke.

[28] Fang Y.N., Tong M.S., Sung P.H., Chen Y.L., Chen C.H., Tsai N.W., Huang C.J., Chang Y.T., Chen S.F., Chang W.N. (2017). Higher neutrophil counts and neutrophil-to-lymphocyte ratio predict prognostic outcomes in patients after non-atrial fibrillation-caused ischemic stroke. Biomed. J..

[29] Yip H.K., Lee M.S., Sun C.K., Chen K.H., Chai H.T., Sung P.H., Lin K.C., Ko S.F., Yuen C.M., Liu C.F. (2017). Therapeutic effects of adipose-derived mesenchymal stem cells against brain death-induced remote organ damage and post-heart transplant acute rejection. Oncotarget.

[30] Florea V.G., Cohn J.N. (2014). The autonomic nervous system and heart failure. Circ. Res..

[31] Korpelainen J.T., Sotaniemi K.A., Myllylä V.V. (1999). Autonomic nervous system disorders in stroke. Clin. Auton. Res..

[32] Al-Qudah Z.A., Yacoub H.A., Souayah N. (2015). Disorders of the Autonomic Nervous System after Hemispheric Cerebrovascular Disorders: An Update. J. Vasc. Interv. Neurol..

[33] Hilz M.J., Moeller S., Akhundova A., Marthol H., Pauli E., De Fina P., Schwab S. (2011). High NIHSS values predict impairment of cardiovascular autonomic control. Stroke.

