# Supplementary material for: The Correlation between Severity of Neurological Impairment and Left Ventricular Function in Patients after Acute Ischemic Stroke
Source: J Clin Med. 2019 Feb 5;8(2):190. doi: 10.3390/jcm8020190 (PMC6406744; doi:10.3390/jcm8020190)
Supplement: Supplementary file 1 [file jcm-08-00190-s001.pdf]

Table S1. Predictors for neurologic impairment with MRS ≥3

Neurological impairment	Univariate			Multivariate		
**Variables**	**OR**	**95% CI**	**P-value**	**OR**	**95% CI**	**P-value**
Age per year	1.060	1.021-1.102	0.003			
Age >in 65 years	3.429	1.484-7.921	0.004	5.254	1.938-14.243	0.001
Male sex	0.596	0.261-1.363	0.220			
Smoker	0.358	0.150-0.851	0.020			
Systolic BP	0.994	0.981-1.008	0.408			
Diastolic BP	0.974	0.951-0.999	0.041			
Hypertension	1.140	0.425-3.063	0.794			
Diabetes	0.750	0.325-1.731	0.500			
Dyslipidemia	0.308	0.134-0.707	0.005	0.269	0.103-0.704	0.007
Old MI	NA	NA	0.999			
Old stroke	1.773	0.612-5.132	0.291			
Atrial fibrillation	3.750	0.766-18.363	0.103			
ACEI or ARB	1.634	0.730-3.659	0.233			
Statin	0.391	0.171-0.894	0.026			
Leukocyte count	0.952	0.821-1.104	0.516			
Hemoglobin	0.956	0.785-1.165	0.657			
Platelet count	0.997	0.991-1.003	0.339			
NLR	1.008	0.951-1.229	0.232			
NLR ≥3.39	2.963	1.144-7.672	0.025			
PLR	1.002	0.998-1.006	0.310			
PLR ≥126	2.297	0.895-5.898	0.084			
NLR ≥3.39 and PLR >126	2.202	0.928-5.225	0.073			
Serum creatinine	0.903	0.596-1.369	0.631			

Total Cholesterol	1.003	0.994-1.011	0.536			
HDL	1.030	0.996-1.066	0.086			
LDL	1.009	0.999-1.019	0.066	1.014	1.003-1.025	0.013
Triglyceride	0.992	0.985-0.998	0.012			
IVS thickness	0.988	0.903-1.081	0.789			
LVEF per %	0.966	0.926-1.006	0.097			
LVEF ≥60%	0.423	0.176-1.018	0.055			
Mild to severe MR	1.005	0.401-2.517	0.992			

Abbreviation: MRS, Modified Rankins Scale; OR, odds ratio; CI, confidence interval; BP, blood pressure; MI, myocardial infarction; ACEI, angiotensin-converting-enzyme inhibitor; ARB, angiotensin II receptor blocker; NLR, neutrophil-to-lymphocyte ratio; PLR, platelet-to-lymphocyte ratio; HDL, high-density lipoprotein; LDL, low-density lipoprotein; IVS, interventricular septum; LVEF, left ventricular ejection fraction; MR, mitral regurgitation

Table S2. Subgroup analysis according to AIS patients with or without AF

**Variables**	**AIS patients with AF (n=11)**	**AIS patients without AF (n=82)**	**P-value**
LVEF (%)	53.35±13.74	66.52±9.58	0.001
MRS	3.73±1.35	2.76±1.49	0.043
NIHSS	13.82±10.34	7.93±8.01	0.029
NLR	5.65±4.80	3.72±3.41	0.094
PLR	183.63±153.30	135.51±124.39	0.241

Abbreviation: AIS = acute ischemic stroke; AF = atrial fibrillation; LVEF = left ventricular ejection fraction; MRS = Modified Rankin Scale; NIHSS, National Institute of Health Stroke Scale; NLR, neutrophil-to-lymphocyte ratio; PLR, platelet-to-lymphocyte ratio
